# Role of Radiation Therapy Differs Between Stages in Primary Bone Large B-Cell Lymphoma in Rituximab Era: A Population-Based Analysis

**DOI:** 10.3389/fonc.2020.01157

**Published:** 2020-07-14

**Authors:** Shengling Ma, Yuanyuan Zhang, Ziying Li, Han Yan, Linghui Xia, Wei Shi, Yu Hu

**Affiliations:** ^1^Institute of Hematology, Union Hospital, Tongji Medical College, Huazhong University of Science and Technology, Wuhan, China; ^2^Department of Oncology, Tongji Hospital, Tongji Medical College, Huazhong University of Science and Technology, Wuhan, China; ^3^Fred Hutchinson Cancer Research Center, Seattle, WA, United States

**Keywords:** primary bone diffuse large B-cell lymphoma, combined modality therapy, radiation, propensity score, nomogram, overall survival, second primary malignancy

## Abstract

**Background:** Primary bone B-cell lymphoma (PB-DLBCL) is a rare entity for which existing data is limited. Whether radiotherapy (RT) should be omitted in the modern treatment of PB-DLBCL is still under debate. We used the SEER database to compare the outcomes among adult patients treated with and without RT in rituximab era.

**Methods:** We included adult patients with PB-DLBCL diagnosed from 2002 to 2016 from SEER 18. The effect of RT on overall survival (OS) using univariate (UVA) and multivariate (MVA) Cox proportional regression and propensity score matching (PSM) was assessed for the entire cohort and subgroups by stages. We calculated the standardized incidence ratio to estimate the short- and long-term risk for second primary malignancies (SPM) from 2002 to 2016 in SEER 18 and 1983–2016 in SEER 9.

**Results:** A total of 1,320 patients were identified, including 856 with early-stage (ES) and 464 with advanced-stage (AS). A decreasing trend was observed in the ES cohort after 2002, while the rate of RT utilization remained stable in the AS cohort over the past three decades. Most patients in ES (63.9%) underwent RT, whereas only 42.2% of AS patients received it. RT significantly improved survival both in UVA and MVA (*P* < 0.001, *P* = 0.010, respectively). PSM analysis further validated the survival advantage of RT (*P* = 0.018). Moreover, a novel web-based prediction model was established to individualize the potential benefit from RT. In subgroup analyses, OS was improved with RT in those who had ES disease (*p* < 0.001) but not in those who had AS disease (*P* = 0.776). With short-term follow up in SEER 18, none of the subgroups showed a significantly elevated risk of developing SPMs. However, RT significantly elevated the late toxicities of second malignancies in ES patients diagnosed at the age of 18–39 or those with appendicular sites of bone involvement.

**Conclusion:** This population-based analysis is the largest PB-DLBCL dataset to date and demonstrates a significant survival benefit associated with RT in early stages rather than advanced stages. In the absence of randomized controlled trials, RT should be considered in ES disease with cautions of second cancers in specific subsets of patients.

## Introduction

Primary lymphoma of bone (PLB) was first reported as a distinct clinical entity by Parker and Jackson in 1984 (1). PLB comprises only 3% of all bone tumors (2) and 5% of all non-Hodgkin lymphomas (3), with diffuse large B-cell lymphomas (DLBCL) being the most common histologic subtype (4, 5). Owing to a large part to its rarity, the limited data on primary bone diffuse large B-cell lymphomas (PB-DLBCL) were mostly from single-centered retrospective studies with limited sample sizes (6, 7) and the role of radiotherapy (RT) has never been investigated by stages, resulting in a vague description of prognostic factors, optimal management, and treatment outcomes.

Although the R-CHOP regimen with anti-CD20 antibody rituximab as first-line treatment has significantly improved the prognosis of patients with DLBCL since 2002 (8), the risk-benefit profile of RT as consolidative therapy remains controversial (9–11). Because PB-DLBCL has unique biological and clinical features (12, 13), traditional R-CHOP regimen is not satisfactory in particular stages. Given that the tumor control with rituximab is not sufficient enough in DLBCL with bone involvement as other sites, combined modality therapy appears to be indicated (6, 13).

RT has been used as a standard treatment modality for PLB with local involvement since the 1960s (2). However, in pre-rituximab era, histology subtypes and clinical entities of PB-DLBCL were often grouped together in a series of small cohort studies, which interfered with objective evaluation the efficacy of RT in PB-DLBCL.

In rituximab era, the recent series of studies have yielded conflicting results. The International Extranodal Lymphoma Study Group (IELSG)-14 study (161 patients) and a study from British Columbia Cancer Agency (103 patients) (5) have shown no survival benefit of consolidative RT after primary chemotherapy (14), whereas a prospective trial (161 patients) of German High-Grade Non-Hodgkin's Lymphoma Study Group (DSHNHL) and another single-center research of the US (102 patients) (6) reported that combined modality therapy (CMT) was associated with better progression-free survival (PFS) and a trend to improve overall survival (OS). These peri-rituximab era trials were limited by the small sample size and the obscure relationship between the efficacy of RT and different stages of PB-DLBCL. With the realization of long-term toxicities of RT, such as a higher rate of second primary malignancies (SPM) of RT (15–17), a concept of omitting the use of radiation therapy was raised in recent years. Considering it would be unfair to exclude it without high-level evidence, we conducted a retrospective study that enclosed the largest samples over the past 3 decades.

Given that early and advanced stages were frequently grouped together in previous studies, and sub-analysis was not performed because of small samples, we took advantage of the modern Surveillance, Epidemiology, and End Results (SEER) database to identify what subset of PB-DLBCL patients may benefit from RT in peri-rituximab era. We also established a dedicated prognostic tool for personalized survival prediction of patients with PB-DLBCL.

## Methods

### Study Population Selection

Based on the third edition of the International Classification of Disease for Oncology (ICDO-3) codes for histology (9,680, 9,684, 9,688) and topography (C40.0–C41.9), we included actively followed-up patients with PB-DLBCL. These patients were excluded: (1) younger than 18 years old (2) diagnosed on autopsy or death certificate (3) with no information on disease stage.

### Definition of Variables

Clinical characteristics of patients included age at diagnosis, sex, race, Ann Arbor stage, the primary site of involvement, survival time, and socioeconomic factors, including marital status, and poverty rate. The Ann Arbor stages were divided into early (Ann Arbor Stage I/II) or advanced (III/IV) stage. The primary site of involvement was classified into 2 categorical variables: Appendicular (C40X) and axial (C41X). Marital status was classified as married (including common law), single (never married), and other (separated/divorced/widowed/unmarried or domestic partner). The percentage of families below poverty in the county of residence drawn from the ACS County Attributes data from 2013 to 2017 was converted into categorical variables according to the interquartile ranges.

### Cohort to Estimate Survival and Treatment Trend: SEER 18, 2002–2016

The SEER 18 registries [1975–2016 varying]) account for the broadest geographic coverage (around 28%) of the U.S. population. The analysis was restricted to adults who were recorded as having received rituximab as part of the first course of treatment after 2002 (8).

The survival curves were generated with the Kaplan–Meier method and compared using the log-rank test. To assess independent prognostic factors, univariate (UVA), and multivariate (MVA) Cox regression analyses were performed.

To further adjust for potential baseline confounders, a propensity score matching (PSM) accounting for all the covariates mentioned above was carried out as described previously (18). In brief, propensity scores were obtained using multivariable logistic regression to estimate the probability of receiving RT. We chose 1:1 fashion with a propensity score radius difference of 0.01 (19), as opposed to many to one matching, to maximize the balance between treatment groups (20). Survival analyses were performed using a Cox proportional hazards model, which were used to compare the survival between the two matched groups.

Moreover, we developed a nomogram and generated a web-based version to individually predict patients' 3-, 5-, and 10-year survival rates. As previously indicated (21–23), two-thirds of the study participants were randomly allocated to a model derivation data set, and one-third were reserved as an independent validation data set. Internal validation was performed by the bootstrap resampling technique, in which regression models were fitted in 500 bootstrap replicates, drawn with replacement from the development sample. External validation was performed with the validation datasets. The nomogram was validated by measuring discrimination and calibration curves both internally (training set) and externally (validation set). Concordance index (C-index) is used to calculate the discrimination between the predicted and real values of Cox models in survival analysis (24). C-index > 0.5 is considered statistically significant, and higher value indicates a stronger predictive ability of the model. Calibration plots exhibit the capability to validate unbiased estimation of outcomes, and an entirely accurate nomogram would result in a plot on which predictions fall along a 45° diagonal line.

### Cohort to Estimate Long-Term Risk for Second Primary Malignancies: SEER 9, 1983–2016

Since SEER 18 registries in MP-SIR/SMR Sessions cover only records after 2000, the long-term incidence of SPM was derived from SEER 9 registries which include data from 1975 to 2016. We limited the analysis from 1983 onward because the Ann Arbor staging system was not available until that time. Secondary cancers were considered if diagnosed more than 2 months after a diagnosis of PB- DLBCL (25). Standardized incidence ratios (SIR) were then calculated as the ratio of the observed (O) to the expected (E) number of cases based on the standard population rates (26).

### Statistical Analysis

All statistical analysis was carried out using the SEERstat 8.3.6, R software version 3.6.3 (http://www.r-project.org) and SPSS version 25 (SPSS Inc, 2016, Armonk, NY). All statistical tests were two-sided with the alpha threshold of significance set at 0.05.

## Results

### Patient Characteristics and Treatment Trend

**Figure 1:**
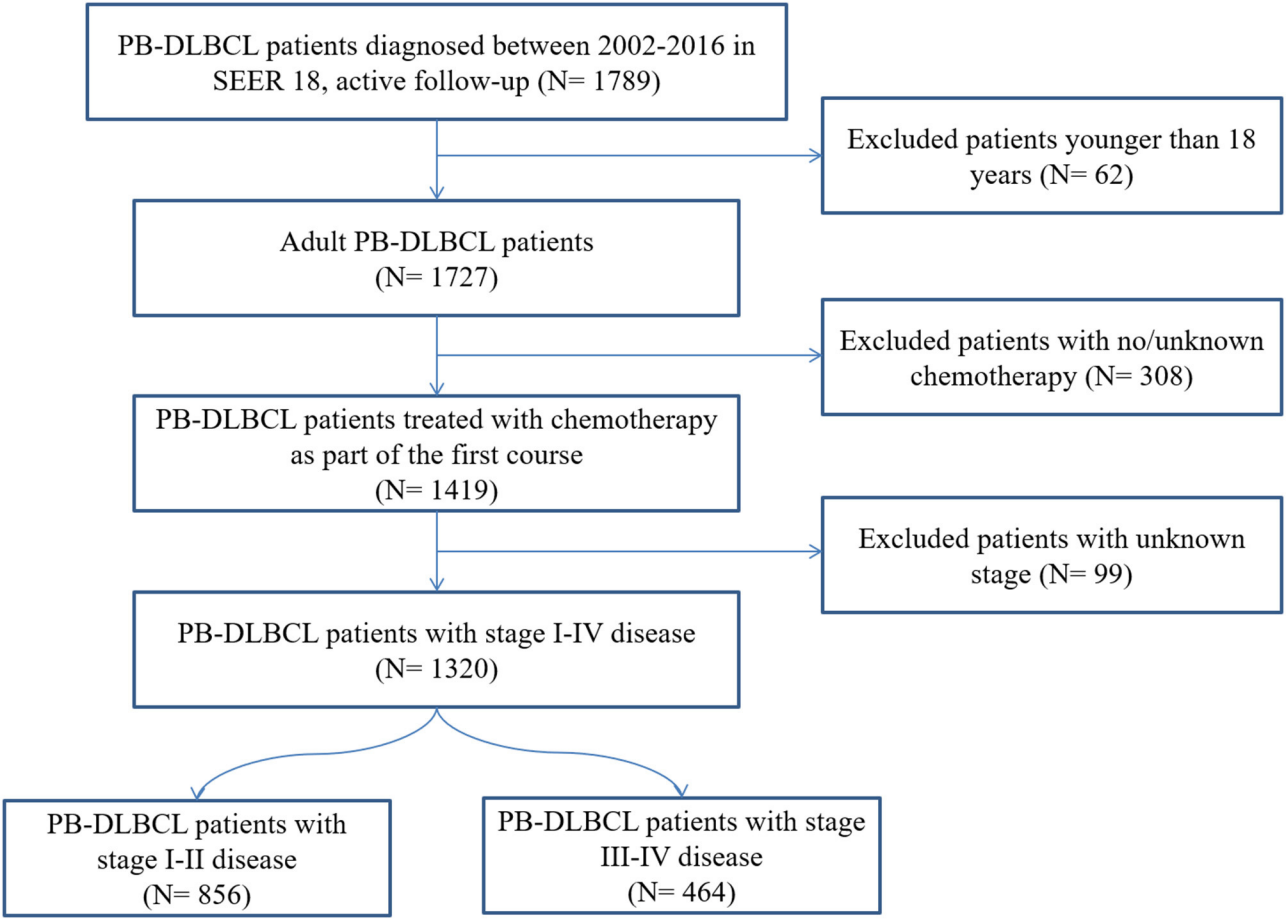
Flow chart for patient selection from the Surveillance, Epidemiology, and End Results (SEER)-18. PB-DLBCL, primary bone diffuse large B-cell lymphomas.

Our SEER 18 query identified 1,320 adults diagnosed with PB-DLBCL and treated with chemotherapy as part of the first course between 2002 and 2016 (Figure 1). Demographic characteristics for patients in the entire cohort are outlined in Table 1. The median age was 61.5 years (range 18–97). The majority of patients were males (54.9%), white (86.8%) with an early-stage predominance (64.9%). The axial bones (63.6%) were more commonly involved than appendicular sites and the majority of patients (743: 56.3%) received CMT in initial treatment.

**Table 1:** Patient characteristics and bias for radiotherapy.

	**Patient characteristics in raw data**				**Patient characteristics after propensity score matching ^≠^**		
**Characteristic**	**Total**	**Chemotherapy alone**	**Combined modality therapy**	P^*^	**Chemotherapy alone**	**Combined modality therapy**	P^*^
	1320	577 (43.7%)	743 (56.3%)		506	506	
Age, y				0.021			0.109
18–39	266 (20.2%)	122 (21.1%)	144 (19.4%)		107 (21.1%)	95 (18.8%)	
40–64	478 (36.2%)	185 (32.1%)	293 (39.4%)		165 (32.6%)	197 (38.9%)	
65+	576 (43.6%)	270 (46.8%)	306 (41.2%)		234 (46.2%)	214 (42.3%)	
Sex				0.121			0.660
Male	725 (54.9%)	303 (52.5%)	422 (56.8%)		259 (51.2%)	266 (52.6%)	
Female	595 (45.1%)	274 (47.5%)	321 (43.2%)		247 (48.8%)	240 (47.4%)	
Year of diagnosis				0.030			0.950
2002–2009	667 (50.5%)	272 (47.1%)	395 (53.2%)		253 (50.0%)	254 (50.2%)	
2010–2016	653 (49.5%)	305 (52.9%)	348 (46.8%)		253 (50.0%)	252 (49.8%)	
Race				0.585			0.500
White	1146 (86.8%)	496 (86.0%)	650 (87.5%)		436 (86.2%)	434 (85.8%)	
Black	99 (7.5%)	44 (7.6%)	55 (7.4%)		37 (7.3%)	45 (8.9%)	
Other	75 (5.7%)	37 (6.4%)	37 (6.4%)		33 (6.5%)	27 (5.3%)	
Stage				<0.001			0.816
I	682 (51.7%)	231 (40.0%)	451 (60.7%)		225 (44.5%)	239 (47.2%)	
II	174 (13.2%)	78 (13.5%)	96 (12.9%)		74 (14.6%)	72 (14.2%)	
III	27 (2.0%)	14 (2.4%)	13 (1.7%)		12 (2.4%)	13 (2.6%)	
IV	437 (33.1%)	254 (44.0%)	183 (24.6%)		195 (38.5%)	182 (36.0%)	
Primary Site				0.977			0.948
Appendicular	481 (36.4%)	210 (36.4%)	271 (36.5%)		185 (36.6%)	184 (36.4%)	
Axial	839 (63.6%)	367 (63.6%)	472 (63.5%)		321 (63.4%)	322 (63.6%)	
Marital status				0.002			0.062
Single	264 (20.0%)	126 (21.8%)	138 (18.6%)		116 (22.9%)	96 (19.0%)	
Married	757 (57.3%)	300 (52.0%)	457 (61.5%)		270 (53.4%)	307 (60.7%)	
Other	299 (22.7%)	151 (26.2%)	148 (19.9%)		120 (23.7%)	103 (20.4%)	
Poverty Rate^§^							0.086
≤ Quartile 1 (6.49%)	334 (25.3%)	130 (22.5%)	204 (27.5%)		116 (22.9%)	111 (21.9%)	
≤ Quartile 2 (9.15%)	329 (24.9%)	128 (22.2%)	201 (27.1%)		116 (22.9%)	142 (28.1%)	
≤ Quartile 3 (13.15%)	280 (28.8%)	184 (31.9%)	196 (26.4%)		170 (33.6%)	138 (27.3%)	
>Quartile 3 (13.15%)	277 (21.0%)	135 (23.4%)	142 (19.1%)		104 (20.6%)	115 (22.7%)	

* *P-value from chi-square tests*.

≠ *Three hundred and eight patients were excluded in the propensity score matching procedure*.

§ *All data are county level*.

**Figure 2:**
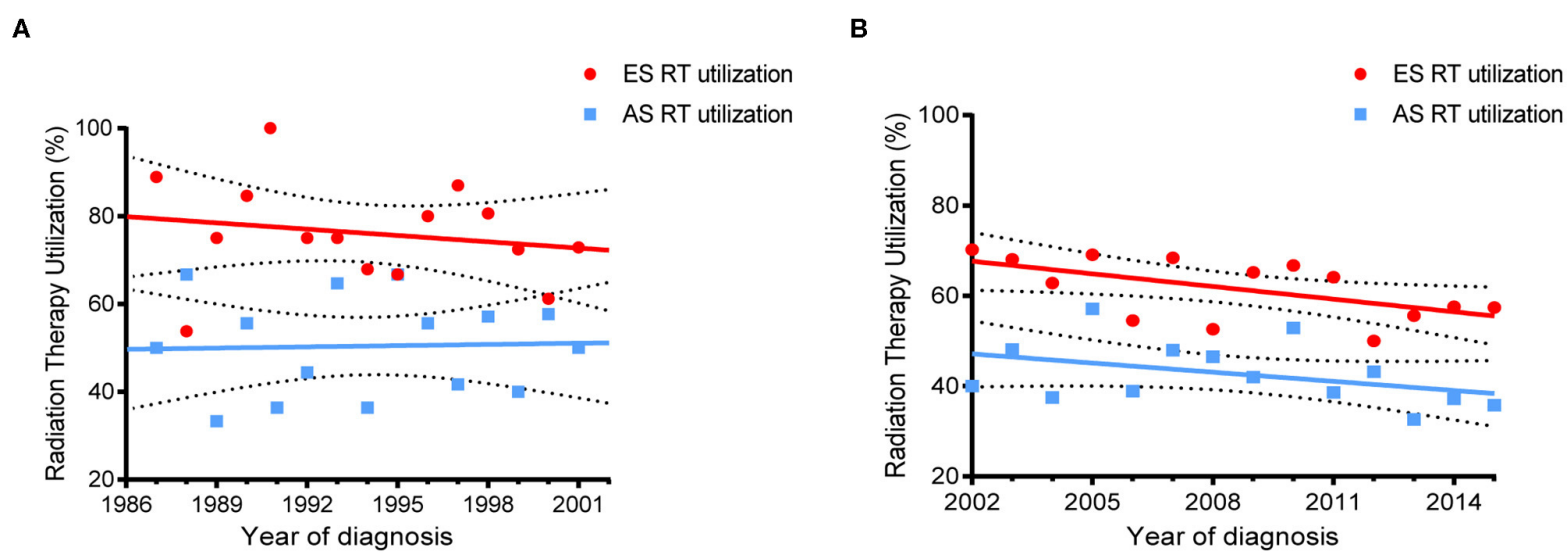
Trends of RT use in PB-DLBCL by different stages. **(A)** For patient diagnosed between 1987–2001; **(B)** For patients diagnosed between 2002–2016.

The rates of RT utilization over time are displayed in Figure 2. For pre-rituximab era, the percent RT utilization by year was stable in both ES and AS patients (slope for the best fit line = −0.4782, *P* = 0.5084 and slope = 0.09214, *P* = 0.8975, respectively). Whereas, RT utilization dramatically decreased in ES (slope = −0.9338, *P* = 0.0316) but didn't change significantly in AS after 2002 (slope = −0.6752, *P* = 0.1498).

The demographic characteristics of the cohort from SEER 9 for estimating the long-term influence on SPM were shown in Table S1. The median follow-up was 72.5 months, with a range of 1–382 months. Of the 701 patients, 470 (67.1%) presented with stage I/II and 231 (32.9%) with stage III/IV. Most of the patients were diagnosed after 2002 (63.2%) and treated with combined modality therapy (66.0%). Among them, 75 patients developed SPM during the observational period. The disease duration from diagnosis of PB-DLBCL till the occurrence of SPM ranged from 3 to 259 months and was 94 months on average.

### Survival and Prognostic Factors

#### Univariate and Multivariate Cox Proportional Hazard Analyses

**Figure 3:**
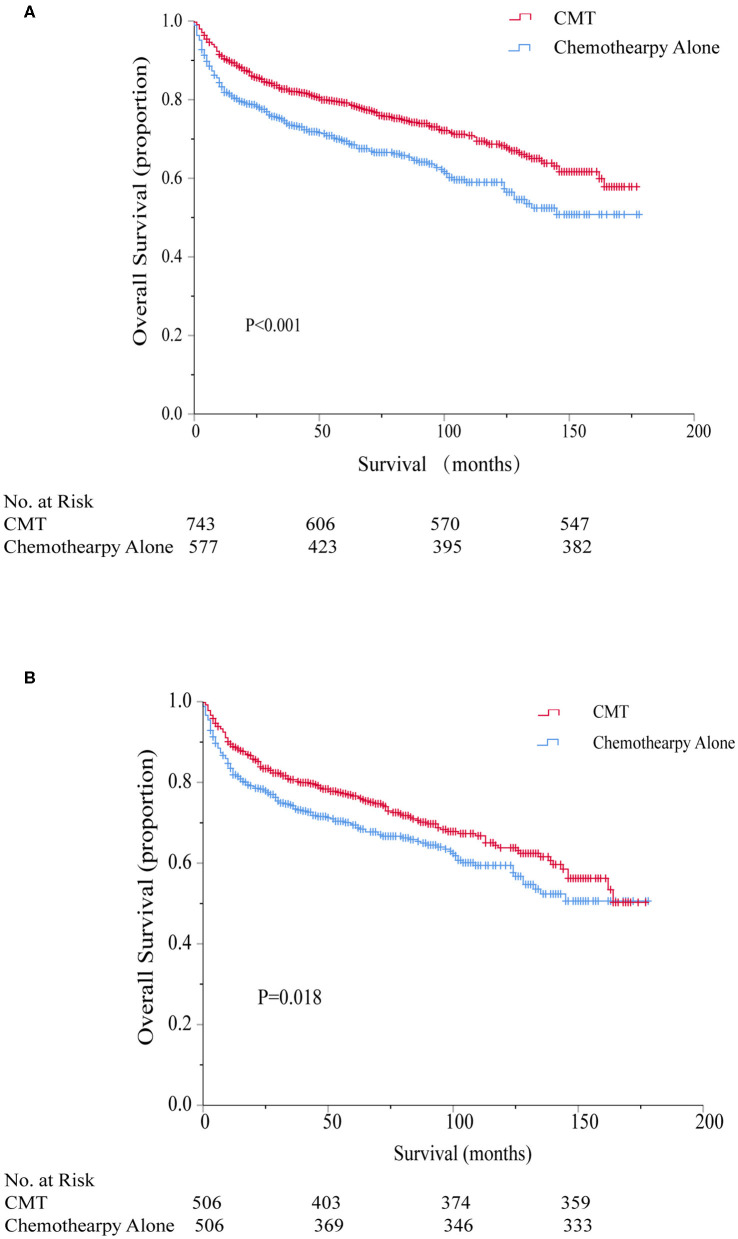
Kaplan–Meier survival comparing combined modality therapy (CMT) vs. chemotherapy alone before **(A)** and after **(B)** propensity score matching. CMT, combined modality therapy.

The 5-year overall survival for the entire cohort was 75%. The consolidation RT resulted in a significant better 5-year OS: 79.2 vs. 69.4%, respectively (HR = 0.66, 95% CI 0.54–0.81, *P* < 0.001). Kaplan–Meier survival curves for CMT and chemotherapy alone treatment groups are depicted in Figure 3A. Univariate survival analyses also demonstrated a worse OS in association with increasing age, stage, axial disease locations, single marital state, and poverty rate. On multivariate analyses, CMT remained a favorable impact on OS (HR = 0.76, 95% CI 0.62–0.94, *P* = 0.010). Older age, increasing stage, primary involvement of axial bones, and higher poverty rate were also independent prognostic factors of worse survival while there was no significant survival difference between different marital statuses after adjusting for other factors (Table 2).

**Table 2:** Prognostic factors for overall survival.

	**Univariate**		**Multivariate**		**Propensity score cox regression**^**†**^	
	**HR (95% CI)**	***P***	**HR (95% CI)**	***P***	**HR (95% CI)**	***P***
Treatment		<0.001		0.010		0.020
Chemotherapy alone	Reference		Reference		Reference	
Combined modality therapy	0.66 (0.54–0.81)		0.76 (0.62–0.94)		0.77 (0.62–0.96)	
Age, y		<0.001		<0.001		<0.001
18–39	Reference		Reference		Reference	
40–64	3.66 (2.19–6.12)		3.60 (2.12–6.10)		3.45 (1.96–6.10)	
65+	10.77 (6.59–17.60)		10.42 (6.22–17.45)		9.87 (5.65–17.25)	
Sex		0.699				0.199
Male	Reference		–		Reference	
Female	1.04 (0.85–1.27)		–		0.86 (0.69–1.08)	
Year of diagnosis		0.468				0.138
2002–2009	Reference		–		Reference	
2010–2016	0.92 (0.74–1.15)		–		0.83 (0.64–1.06)	
Race		0.728				0.624
White	Reference		–		Reference	
Black	0.87 (0.59–1.28)		–		1.23 (0.80–1.90)	
Other	1.07 (0.69–1.64)		–		1.08 (0.65–1.78)	
Stage		<0.001		<0.001		0.004
I	Reference		Reference		Reference	
II	1.20 (0.87–1.65)		1.25 (0.91–1.73)		1.15 (0.80–1.64)	
III	1.72 (0.91–3.26)		1.36 (0.71–2.59)		1.46 (0.74–2.90)	
IV	1.86 (1.50–2.31)		1.63 (1.31–2.04)		1.57 (1.23–2.01)	
Primary Site		<0.001		<0.001		0.002
Appendicular	Reference		Reference		Reference	
Axial	2.01 (1.60–2.54)		1.57 (1.24–1.98)		1.51 (1.17–1.96)	
Marital status		<0.001		0.831		0.527
Single	Reference		Reference		Reference	
Married	1.76 (1.30–2.40)		0.90 (0.66–1.25)		0.91 (0.63–1.30)	
Other	2.32 (1.66–3.26)		0.92 (0.65–1.32)		1.05 (0.70–1.57)	
Poverty Rate^§^		0.017		0.001		0.036
≤ Quartile 1 (6.49%)	Reference		Reference		Reference	
≤ Quartile 2 (9.15%)	1.06 (0.79–1.42)		1.04 (0.78–1.39)		0.94 (0.68–1.30)	
≤ Quartile 3 (13.15%)	1.11 (0.84–1.47)		1.05 (0.79–1.39)		1.02 (0.75–1.40)	
>Quartile 3 (13.15%)	1.51 (1.14–2.01)		1.61 (1.22–2.15)		1.44 (1.04–2.01)	

† *Full model multivariable cox regression analysis after propensity score matching*.

§ *All data are county level*.

#### Propensity Score Matching

To further account for potential bias attributing to the imbalance between the CMT and chemotherapy alone groups, propensity score matching was performed to optimally adjust for the imbalance between the regarding all baseline variables. As shown in Table 1, imbalance across groups was avoided for all included parameters after propensity score matching. Radiation utilization was confirmed to be a significant protective predictor for overall survival even after PSM (HR = 0.77, 95% CI 0.62–0.96, *P* = 0.018) and the associated Kaplan–Meier survival curves for the PSM analysis is displayed in Figure 3B.

#### Development and Validation of a Prognostic Nomogram

**Figure 4:**
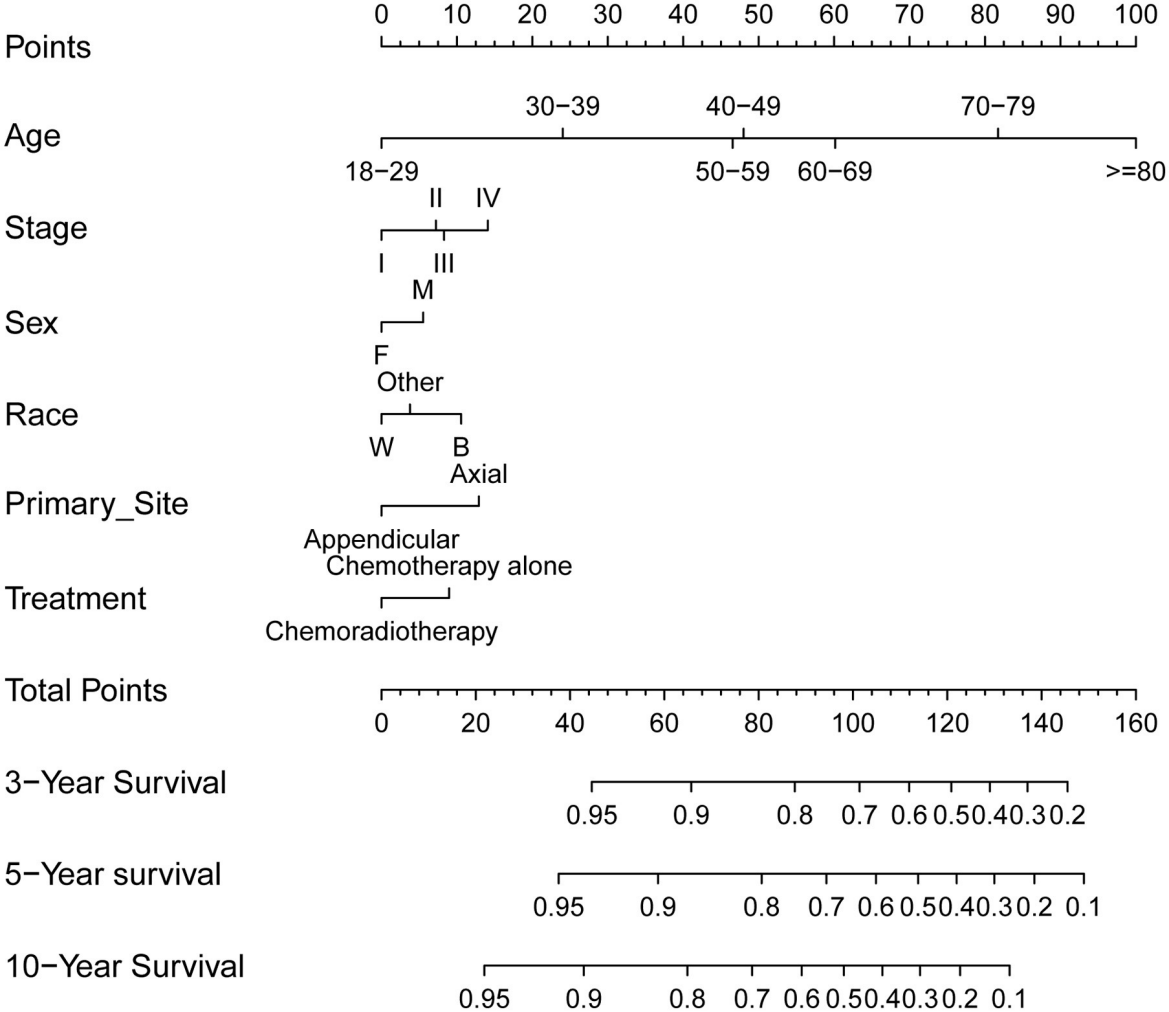
Prognostic nomogram (A) to predict 3-/5-/10-year overall survival in PB-DLBCL patients. F, female; M, male; W, white; B, black; CMT, combined modality therapy.

Furthermore, to predict 3-, 5-, and 10-year OS for PB-DLBCL patients, a nomogram was developed including significant indicators (Figure 4). Points are assigned based on the hierarchy of effects on OS. Accuracy of the nomogram was examined using C-index and calibration plot with both training and validation cohorts. The C-index on internal and external validations presented values of 0.74 and 0.76, respectively, revealing excellent performance in predicting the prognosis of patients with PB-DLBCL. Notably, the data points in internal and external calibration plots fall close to this line in calibration plots, showing high consistency between predicted and actual observed 3-, 5-, and 10-year OS for PB-DLBCL patients (Figure S1).

#### Development of Webserver for Convenient Access to Nomogram

**Figure 5:**
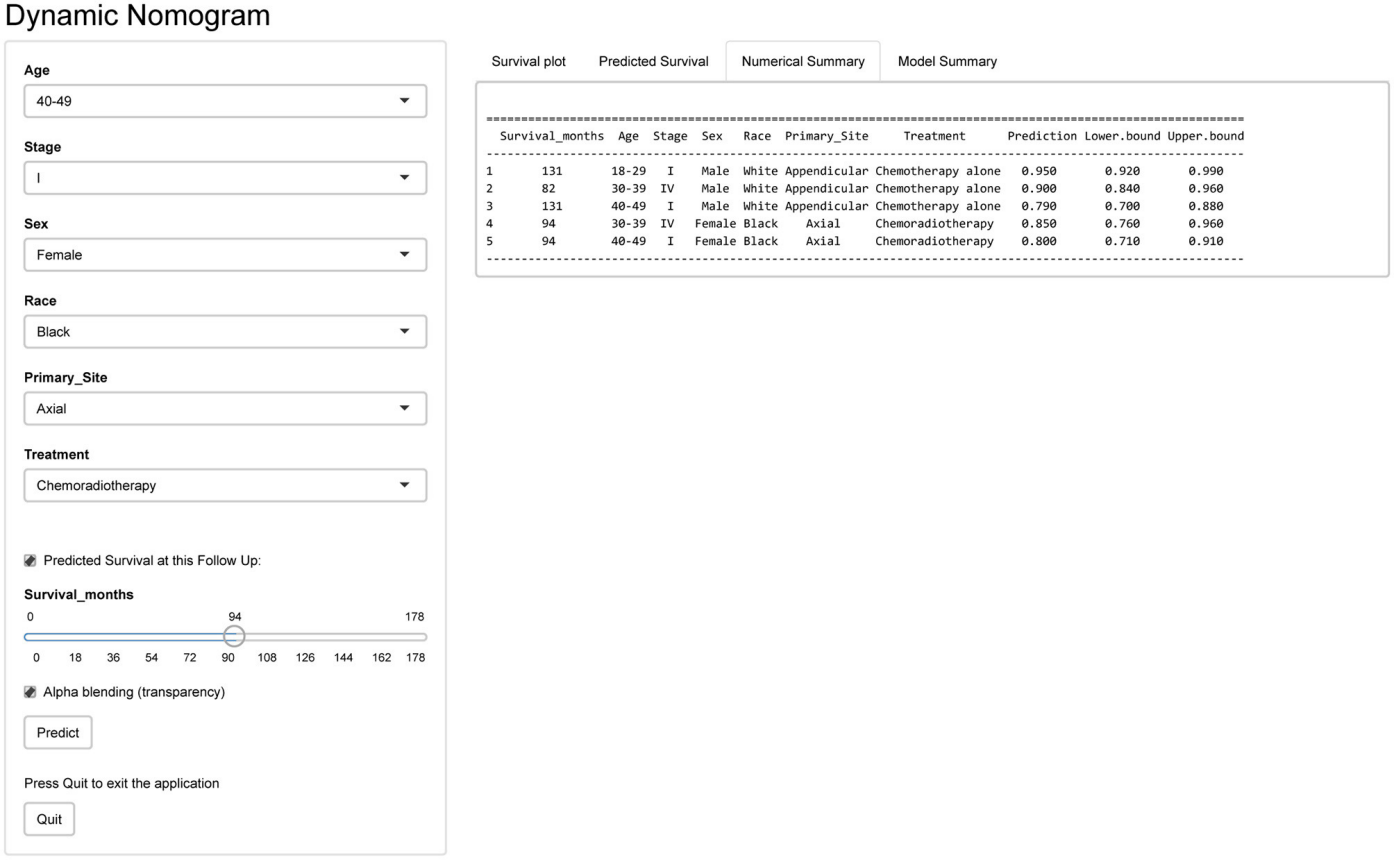
Snapshot of the webserver for nomogram.

An online version of our nomogram (Figure 5) can be accessed at https://pbdlbcl.shinyapps.io/PB-DLBCL/ to assist researchers and clinicians. Predicted survival probability and its 95% confidence interval across time can be easily determined by inputting clinical features and reading output figures and tables generated by the webserver.

### Role of RT in Early-Stage Patients

**Figure 6:**
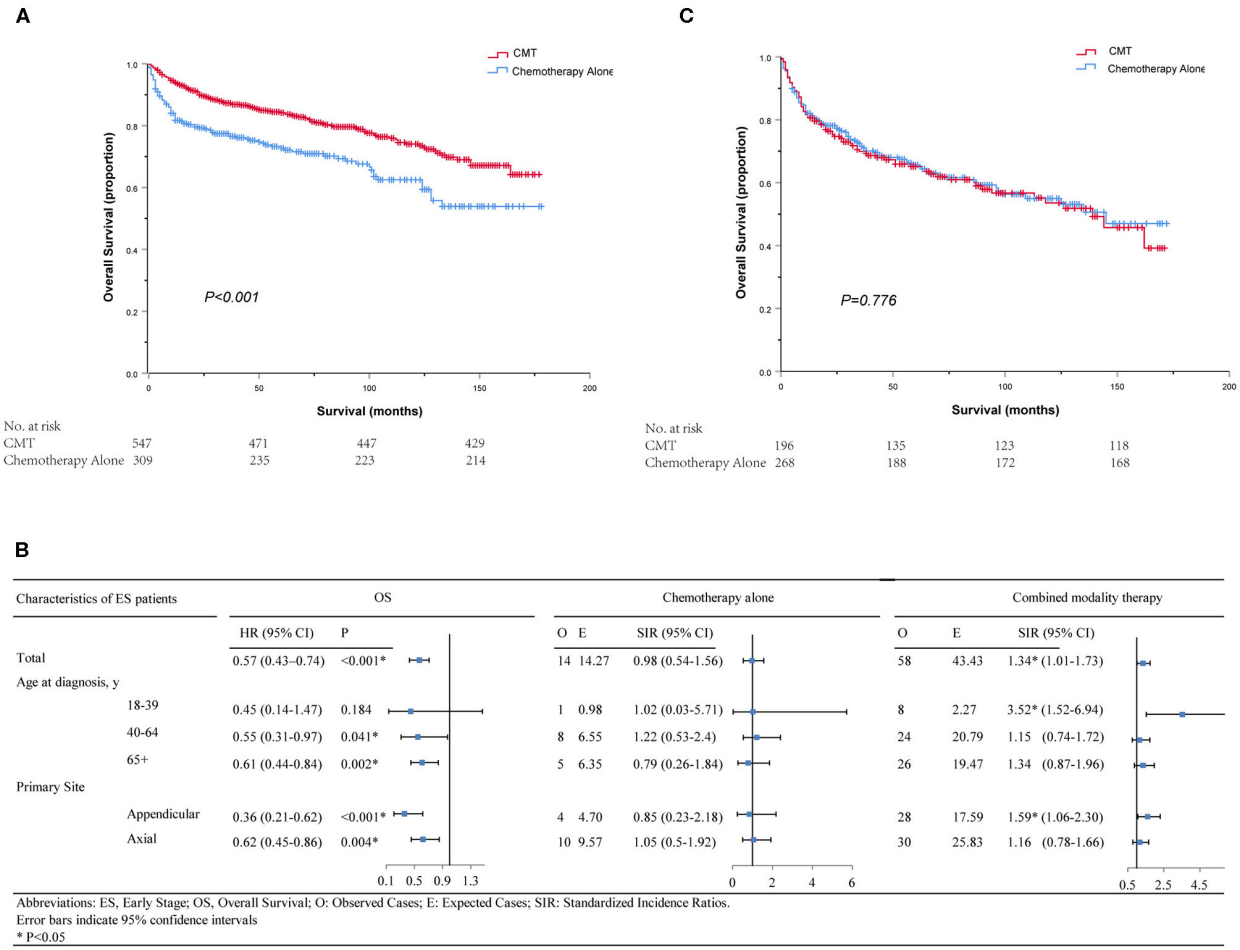
Kaplan–Meier survival in patients with stage I–II **(A)** and stage III–IV **(C)**; Subgroup analyses of survival and secondary primary cancers by age, primary site and treatment in patients with early-stage PB-DLBCL **(B)**. CMT, combined modality therapy.

A total of 856 patients were diagnosed with stage I-II PB-DLBCL, with a median follow-up of 64 months (range 0–178). Most of them (63.9%) received CMT as the first-line treatment (Table S2). The survival impact of primary radiotherapy for patients with limited-stage disease is outlined in Figure 6A. On univariate analysis (Table S3), CMT was significantly associated with prolonged OS (5-year OS = 84.2%, HR = 0.57, 95% CI = 0.43–0.74, *p* < 0.001) compared to chemotherapy alone (5-year OS = 72.7%) as shown in Figure 3A. In adjusted multivariate Cox model, radiotherapy, age at diagnosis, primary site, poverty rate remained independent prognostic factors for both OSs.

By propensity score matching, imbalance in potential baseline confounders across the two treatment groups could be avoided for most patient- and treatment-related factors, except for the poverty rate (Table S2). CMT was still significantly in correlation with better OS (HR = 0.62, 95% CI = 0.44–0.86, *p* = 0.004) and the survival curves calculated using the Kaplan-Meier method were displayed in Figure S2. Diagnosis at younger ages, primary involvement in appendicular sites and lower poverty rate were associated with better OS in both groups, consistent with the results of the Cox model mentioned above (Table S3).

We further determined the risk-benefit ratio of including RT as part of the primary treatment in different subsets for early-stage patients. In total, there were 54 ES patients who developed SPMs after PB-DLBCL and the histology subtypes were summarized in Table S4. Furthermore, subgroup analyses showed that young adults aged between 18 and 39 years did not achieve overall survival benefit from RT but suffered from more SPMs (SIR = 3.52, 95% CI 1.52–6.94, P < 0.05) according to the long-term follow-up results of SEER 9 (Figure 6). As depicted in Table S5, the relative risks of developing subsequent solid malignancies of the ascending colon, respiratory system, bones, and joints were significantly increased after RT treatment. Additionally, there was an 18.57-fold significant increase in the relative risk of subsequent cancers of the tonsil for middle-aged patients treated with CMT (SIR = 18.57, 95% CI 2.25–67.09, *P* < 0.05). However, none of the subgroup analyses showed a significantly elevated risk of SPMs in SEER 18 between 2002 and 2016.

For site-specific SPM stratified by primary site of bone involvement, it is noteworthy that although benefit of OS was observed in patients with the appendicular site of bone involvement, the overall SIR for SPM following PB-DLBCL was significantly elevated (SIR = 1.59, 95% CI 1.06–2.3, *P* < 0.05, Figure 6B). For PB-DLBCL patients with appendicular bone involvement, SIRs were significantly higher for bone and joint cancers (48-fold), uterus cancers (90-fold) and hematological malignancies (3-fold) following CMT (Table S6).

### Role of RT in Advanced-Stage Patients

For the 464 patients who presented with AS, the median clinical follow-up period was 45 months, ranging from 0 to 172. In contrast with stage I-II, a minority of patients (42.2%) in stage III-IV underwent CMT as their first treatment course (Table S7). Surprisingly, neither univariate nor multivariate analysis revealed any significant association between additional RT and survival benefits (5-year OS = 65.1% for CMT and 65.7% for chemotherapy alone, *p* = 0.776, Figure 6C). Only age at diagnosis and primary site of cancer involvement was independently associated with OS in both groups (Table S8). Since all variables were well-balanced without significant differences between the CMT and chemotherapy subgroups, PSM was not performed for this cohort. Besides, there was no significant increase in SPM risk in neither RT nor no-RT group for AS patients in SEER 18 or SEER 9 database.

## Discussion

To the best of our knowledge, the present study is the largest PB-DLBCL cohort to date, as well as the first population-based study using propensity score matching and individualized prediction tools to clarify the impact of consolidation RT for patients with PB-DLBCL in rituximab era. Our investigation yields 3 key results: First, linear regressions revealed a significant decrease in the rate of patients with early-stage PB-DLBCL undergoing primary RT since 2002, but the proportion of RT utilization in advanced-stage patients remained lower and stable. Moreover, a clear association of chemoradiotherapy in stage I-II patients with decreased overall mortality in a large patient cohort whereas no significant difference in stage III–IV was detected, even after adjusting in multivariable or propensity score analyses. Furthermore, the utilization of RT in early-stage PB-DLBCL may predispose young adults (18–39 years) and primary appendicular skeletal involved patients to the long-term risk of secondary malignancies.

While the addition of rituximab in traditional chemotherapy has been widely adopted with promising results, whether radiation should be omitted has become a controversial focus. Four large randomized trials have explored the role of consolidation RT for stage I-II non-Hodgkin lymphoma, but were performed in the pre-rituximab era (27–30). After the introduction of rituximab, a large retrospective study demonstrated the benefit of consolidative RT plus R-CHOP chemotherapy in stage I–II DLBCL rather than in stage III–IV and that patients with and without bulky disease benefited equally from RT (11). Radiation as a consolidative therapy to initial bulky site after chemotherapy has been associated with improved outcomes in patients with aggressive B-cell lymphoma (31, 32). Retrospective research on primary bone lymphoma which mainly consisted of diffuse large B-cell histology subtype also indicated that patients who have undergone CMT have a more favorable outcome (2, 33–35). The UNFOLDER randomized trial by the German High-Grade Non-Hodgkin's Lymphoma Study Group (DSHNHL) included 450 patients receiving either R-CHOP-14 or R-CHOP-21, patients with extranodal or bulky disease were randomized to add RT or not. The 2 RT arms were closed when a second interim analysis showed a higher failure rate in the no-RT arm. A RICOVER-noRTh study (31) found that among patients with bulky disease, the addition of consolidation RT improved PFS and OS.

PB-DLBCL is a distinct clinicopathologic entity, and a germinal-center-like immunophenotype characterizes roughly half of large B-cell lymphomas of bone and biologically accounted for the favorable prognosis (12, 36). There is a general paucity of information available for consolidative RT in patients with PB-DLBCL and several studies focusing on this issue indicated different conclusions (Table 3). Consistent to our investigation, a clear survival benefit of consolidative RT was most recently demonstrated in a single-centered study conducted by Tao et al. (6) in PB-DLBCL patients with stage I-II disease (5-years OS, 97 vs. 67%, *P* = 0.0007) and PFS (5-years OS, 94 vs. 74%, *P* = 0.03) whereas addition of RT did not affect survival outcomes for patients with stage III-IV disease. Besides, they also found that bulky disease of >5 cm or >7.5 cm was not associated with worse PFS or OS. However, in a subset analysis of the German High-Grade Non-Hodgkin's Lymphoma Study Group (DSHNHL) prospective trials (13), although RT was an independent factor in the multivariable analysis in the overall analysis, benefit could hardly be found when patients were grouped by stage: for 78 patients with early-stage diseases, the HR was 0.4 (*P* = 0.146) for EFS and 1.2 (*P* = 0.864) for OS; for 83 patients with advanced-stage disease, the HRs were 0.3 (*P* = 0.001) for EFS and 0.4 (*P* = 0.059) for OS, respectively. Unlike our study, 73.3% of analyzed patients had been treated without rituximab, which may result in unbalanced comparisons and low-level evidence to modern era. Another retrospective study from the British Columbia Cancer Agency included 80 advanced-stage patients with primary bone DLBCL (5). This is one of the few studies that reported a worse survival from CMT than chemotherapy alone for advanced stage (25 vs. 56%, *P* = 0.025). However, their findings must be interpreted with caution because most treatment modalities were conducted in pre-positron emission tomography (PET) and pre-rituximab era. Even though the discretionary nature of the indications for bone irradiation and heterogeneity of bone involvement (bulky or not, single or multiple) preclude firm conclusions concerning additive RT in advanced-stage PB-DLBCL, a significant palliative role and improved local control should be considered for aggressive lymphoma causing local symptoms in patients who were refractory to best chemotherapy or transplant maneuver or sometimes as salvage therapy when disease recurs (37–39).

**Table 3:** Recent PB-DLBCL Series comparing CMT and chemotherapy alone.

**Study**	**No. of patients**	**Percentage of bulky disease: No RT vs. RT**	**Age, y**	**Study type**	**Years**	**Finding**
Current study	1,320 (Stages I, II: 856; Stage III–IV: 464)	NA	Adults	US population-based SEER database	2002–2016	Significantly improved OS with RT in stage I–II, but not in III–IV
(6)	102 (All stages)	>7.5 cm: 28.6 vs. 28.4%	16–87 y	The University of Texas MD Anderson Cancer Center (single institution)	1988–2013	Significantly improved OS and PFS with RT in stage I–II, but not in III–IV. Bulky disease of >5 cm or >7.5 cm was not associated with worse PFS or OS.
(14)	161 (Stage I and II)	>10 cm: 23% overall	Adults	The International Extranodal Lymphoma Study Group (IELSG)-14 study	1980–2005	The addition of RT was not associated with better OS and PFS.
(13)	161 (Stages I, II: 78; Stage III–IV: 83)	Bulky: 17.9 vs. 33.8%	Adults	The German High-Grade Non-Hodgkin's Lymphoma Study Group	1993–2005	RT Significantly improved EFS but not OS as a whole. No significant benefit OS was observed in the subgroup of stage I–II or III–IV. Bulky disease was associated with worse OS but not PFS.
(5)	80 (Stage IV or stage IE or IIE disease plus either B symptoms or tumor ≥10 cm in maximum diameter)	NA	Adults	The British Columbia Cancer Agency	1983–2005	The addition of RT was associated with worse OS.

*SEER, surveillance, epidemiology, and end results program; RT, radiotherapy; OS, overall survival; PFS, progression-free survival; EFS, event-free survival; NA, not applicable*.

Consistent with the previous studies for early-stage DLBCL (10, 40), the decreasing trend of RT utilization in PB-DLBCL patients over the past 3 decades might be associated with the increasing concerns of radiation-related toxicities (41–43). Partly owing to the short-term follow up from 2002 to 2016, none of the subgroups showed elevated risks of developing SPMs. When it comes to the late toxicities of RT, no significantly higher SPM was observed in AS patients in the SEER 9 cohort, which is consistent with the previous reports (44–46). Nevertheless, for ES patients, a higher risk of SPM was observed in patients with primary appendicular bone involvement and those aged from 18 to 39 years. This result may be partly explained by the fact that localized stage, younger age, and appendicular skeleton involvement were associated with dramatically prolonged survival time. Consequently, host susceptibility, shared etiological elements, additional treatments, and other exposures, and enhanced clinical surveillance (47) may lead to the occurrence of SPMs, an important cause of morbidity and mortality (43, 48). Another possible explanation for this is that ES DLBCL may be biologically distinct from AS DLBCL. Roberts et al. (49) demonstrated that ES DLBCL was significantly more likely to belong to germinal center origin compared with AS disease, and Stephens et al. (50) suggested that increased late relapses in ES compared with AS disease may result from biological differences. Therefore, it is plausible that the genetic basis of oncogenesis in ES DLBCL predisposes to distinct SPMs, and that these SPMs may share common genetic origins with ES DLBCL (51).

Other groups have sought to individualize treatment strategies for DLBCL using an interim positron emission tomography (PET)-adapted approach in the modern era (52, 53). Post-chemotherapy FDG-PET/CT can help precisely identify responders and avoid excrescent radiotherapy (54, 55). As a result, patients who are PET-positive following chemotherapy are more likely to receive and benefit from consolidation radiotherapy (56, 57). Besides, we observed that a higher poverty rate was associated with worse OS for the residents. This result may be explained by the fact that the weakness in economic strength may be related to a lower likelihood of PET utilization (58). Although PET-scans have been demonstrated for the detection of DLBCL involving bone with a high sensitivity (59), careful interpretation of residual positivity in the skeleton is recommended because causes other than persistent lymphoma such as bone healing or inflammation may contribute to false-positive cases (60).

Analyzing rare malignancies like PB-DLBCL by querying nationwide datasets has been advocated in settings for which there is a paucity of prospective data or trials (61). Due to linkage with the mandatory national cancer and death registries, these data usually have high completeness and represent the entire patient population. Nonetheless, several inherent limitations should be acknowledged in this study. The main drawback is the lack of records about radiation doses and fields as well as chemotherapy agents in the SEER database. As a result, the included patients may not 100% undergo R-CHOP and RT may not be consistently directed to the primary lesion in those with advanced disease. Finally, although we carried out risk-adjust using both multivariable and propensity score analyses for potential baseline confounders, unavailable prognostic factors such as the presence of bulky disease, the risk of relapse, performance status, International Prognostic Index were not adjusted in the study.

In conclusion, the present study supports the favorable impact of consolidation RT on overall survival in patients with stage I-II at initial diagnosis. Nonetheless, the radiation-induced second malignancies should not be neglected and call for considerable attention in young adults and patients with appendicular bone involvement. For patients with advanced-stage, irradiation is not associated with better outcomes or higher risk for secondary cancers but should be reserved predominantly for patients who present with bulky disease. Our findings and the first user-friendly nomogram based on large series will help clinicians to predict the prognosis, choose optimal treatments, and guide individualized follow-up management for patients with PB-DLBCL.

## Data Availability Statement

Publicly available datasets were analyzed in this study. This data can be found here: Surveillance, Epidemiology, and End Results (SEER) database (https://seer.cancer.gov/).

## Author Contributions

SM and YZ collected and analyzed the data. ZL and HY contributed to data analysis and the initial draft of the manuscript. WS edited the paper. YH revised the paper. All authors reviewed the paper and approved the final manuscript.

## Conflict of Interest

The authors declare that the research was conducted in the absence of any commercial or financial relationships that could be construed as a potential conflict of interest.
