# Supplementary material for: Role of Radiation Therapy Differs Between Stages in Primary Bone Large B-Cell Lymphoma in Rituximab Era: A Population-Based Analysis
Source: Front Oncol. 2020 Jul 14;10:1157. doi: 10.3389/fonc.2020.01157 (PMC7372636; doi:10.3389/fonc.2020.01157)
Supplement: Supplementary file 1 [file Data_Sheet_1.docx]

Table S1: Patient Characteristics of Cohort from SEER 9 Database.

| Characteristic | Total | Chemotherapy alone | Combined modality therapy | P* |
| --- | --- | --- | --- | --- |
|  | 701(100.0%) | 238(34.0%) | 463(66.0%) |  |
| Age, y |  |  |  | 0.614 |
| 18-39 | 159(22.7%) | 54(22.7%) | 105(22.7%) |  |
| 40-64 | 287(40.9%) | 92(38.7%) | 195(42.1%) |  |
| 65+ | 255(36.4%) | 92(38.7%) | 163(35.2%) |  |
| Sex |  |  |  | 0.112 |
| Male | 406(57.9%) | 128(53.8%) | 278(60.0%) |  |
| Female | 295(42.1%) | 110(46.2%) | 185(40.0%) |  |
| Year of diagnosis |  |  |  | 0.113 |
| 1983-2001 | 258(36.8%) | 78(32.8%) | 180(38.9%) |  |
| 2002-2016 | 443(63.2%) | 160(67.2%) | 283(61.1%) |  |
| Race |  |  |  | 0.314 |
| White | 616(87.9%) | 209(87.8%) | 407(87.9%) |  |
| Black | 49(7.0%) | 20(8.4%) | 29(6.3%) |  |
| Other | 36(5.1%) | 9(3.8%) | 27(5.8%) |  |
| Stage |  |  |  | <0.001 |
| I | 381(54.4%) | 95(39.9%) | 286(61.8%) |  |
| II | 89(12.7%) | 27(11.3%) | 62(13.4%) |  |
| III | 13(1.9%) | 4(1.7%) | 9(1.9%) |  |
| IV | 218(31.1%) | 112(47.1%) | 106(22.9%) |  |
| Primary Site |  |  |  | 0.019 |
| Appendicular | 281(40.1%) | 81(34.0%) | 200(43.2%) |  |
| Axial | 420(59.9%) | 157(66.0%) | 263(56.8%) |  |
| Marital status |  |  |  | 0.141 |
| Married | 417(59.5%) | 130(54.6%) | 287(62.0%) |  |
| Single | 130(18.5%) | 47(19.7%) | 83(17.9%) |  |
| Other | 154(22.0%) | 61(25.6%) | 93(20.1%) |  |

* P-value from chi-square tests.

Table S2: Patient Characteristics and Bias for Radiotherapy in Early-stage Patients.

|  | Patient Characteristics in Raw Data | | | |  | Patient Characteristics After Propensity Score Matchingǂ | | |
| --- | --- | --- | --- | --- | --- | --- | --- | --- |
| Characteristic | Total | Chemotherapy alone | Combined modality therapy | P* |  | Chemotherapy alone | Combined modality therapy | P* |
|  | 856 | 309(36.1%) | 547(63.9%) |  |  | 66 | 68 |  |
| Age, y |  |  |  | 0.052 |  |  |  | 0.192 |
| 18-39 | 187(21.8%) | 70(22.7%) | 117(21.4%) |  |  | 66(22.9%) | 68(23.6%) |  |
| 40-64 | 313(36.6%) | 97(31.4%) | 216(39.5%) |  |  | 91(31.6%) | 109(37.8%) |  |
| 65+ | 356(41.6%) | 142(46.0%) | 214(39.1%) |  |  | 131(45.5%) | 111(38.5%) |  |
| Sex |  |  |  | 0.029 |  |  |  | 0.242 |
| Male | 469(54.8%) | 154(49.8%) | 315(57.6%) |  |  | 149(51.7%) | 163(56.6%) |  |
| Female | 387(45.2%) | 155(50.2%) | 232(42.4%) |  |  | 139(48.3%) | 125(43.4%) |  |
| Year of diagnosis |  |  |  | 0.034 |  |  |  | 0.504 |
| 2002-2009 | 429(50.1%) | 140(45.3%) | 289(52.8%) |  |  | 137(47.6%) | 129(44.8%) |  |
| 2010-2016 | 427(49.9%) | 169(54.7%) | 258(47.2%) |  |  | 151(52.4%) | 159(55.2%) |  |
| Race |  |  |  | 0.391 |  |  |  | 0.694 |
| White | 738(86.2%) | 260(84.1%) | 478(87.4%) |  |  | 246(85.4%) | 239(83.0%) |  |
| Black | 70(8.2%) | 30(9.7%) | 40(7.3%) |  |  | 27(9.4%) | 30(10.4%) |  |
| Other | 48(5.6%) | 19(6.1%) | 29(5.3%) |  |  | 15(5.2%) | 19(6.6%) |  |
| Stage |  |  |  | 0.007 |  |  |  | 0.191 |
| I | 682(79.7%) | 231(74.8%) | 451(82.4%) |  |  | 218(75.7%) | 231(80.2%) |  |
| II | 174(20.3%) | 78(25.2%) | 96(17.6%) |  |  | 70(24.3%) | 57(19.8%) |  |
| Primary Site |  |  |  | 0.166 |  |  |  | 0.674 |
| Appendicular | 342(40.0%) | 133(43.0%) | 209(38.2%) |  |  | 121(42.0%) | 126(43.8%) |  |
| Axial | 514(60.0%) | 176(57.0%) | 338(61.8%) |  |  | 167(58.0%) | 162(56.2%) |  |
| Marital status |  |  |  | 0.010 |  |  |  | 0.454 |
| Single | 187(21.8%) | 78(25.2%) | 109(19.9%) |  |  | 73(25.3%) | 65(22.6%) |  |
| Married | 496(57.9%) | 158(51.1%) | 338(61.8%) |  |  | 151(52.4%) | 166(57.6%) |  |
| Other | 173(20.2%) | 73(23.6%) | 100(18.3%) |  |  | 64(22.2%) | 57(19.8%) |  |
| Poverty Rate§ |  |  |  | ＜0.001 |  |  |  | 0.004 |
| ≤Quartile 1 (6.49%) | 218(25.5%) | 60(19.4%) | 158(28.9%) |  |  | 60(20.8%) | 52(18.1%) |  |
| ≤Quartile 2 (9.15%) | 209(24.4%) | 56(18.1%) | 153(28.0%) |  |  | 56(19.4%) | 74(25.7%) |  |
| ≤Quartile 3 (13.15%) | 242(28.3%) | 110(35.6%) | 132(24.1%) |  |  | 107(37.2%) | 73(25.3%) |  |
| >Quartile 3 (13.15%) | 187(21.8%) | 83(26.9%) | 104(19.0%) |  |  | 65(22.6%) | 89(30.9%) |  |

* P-value from chi-square tests.
ǂ Seven hundred and twenty-two patients were excluded in the propensity score matching procedure.
§ All data are county level.

Table S3: Prognostic Factors for Overall Survival in Early-stage Patients.

|  | Univariate | |  | Multivariate | |  | Propensity Score Cox Regression† | |
| --- | --- | --- | --- | --- | --- | --- | --- | --- |
|  | HR (95% CI) | P |  | HR (95% CI) | P |  | HR (95% CI) | P |
| Treatment |  | <0.001 |  |  | <0.001 |  |  | 0.004 |
| Chemotherapy alone | Reference |  |  | Reference |  |  | Reference |  |
| Combined modality therapy | 0.56(0.43-0.74) |  |  | 0.59(0.44-0.78) |  |  | 0.62(0.44-0.86) |  |
| Age, y |  | <0.001 |  |  | <0.001 |  |  | <0.001 |
| 18-39 | Reference |  |  | Reference |  |  | Reference |  |
| 40-64 | 2.88(1.50-5.53) |  |  | 3.14(1.59-6.19) |  |  | 2.70(1.28-5.70) |  |
| 65+ | 10.66(5.77-19.69) |  |  | 11.06(5.74-21.33) |  |  | 9.39(4.54-19.44) |  |
| Sex |  | 0.589 |  |  |  |  |  | 0.207 |
| Male | Reference |  |  | - |  |  | Reference |  |
| Female | 1.08(0.82-1.41) |  |  | - |  |  | 0.81(0.58-1.13) |  |
| Year of diagnosis |  | 0.468 |  |  |  |  |  | 0.825 |
| 2002-2009 | Reference |  |  | - |  |  | Reference |  |
| 2010-2016 | 1.00(0.74-1.36) |  |  | - |  |  | 0.96(0.67-1.38) |  |
| Race |  | 0.782 |  |  |  |  |  | 0.875 |
| White | Reference |  |  | - |  |  | Reference |  |
| Black | 0.84(0.50-1.39) |  |  | - |  |  | 1.16(0.66-2.05) |  |
| Other | 1.03(0.56-1.89) |  |  | - |  |  | 1.00(0.46-2.19) |  |
| Stage |  | 0.279 |  |  |  |  |  | 0.428 |
| I | Reference |  |  | - |  |  | Reference |  |
| II | 1.19(0.87-1.65) |  |  | - |  |  | 1.17(0.79-1.72) |  |
| Primary Site |  | <0.001 |  |  | 0.001 |  |  | 0.015 |
| Appendicular | Reference |  |  | Reference |  |  | Reference |  |
| Axial | 2.19(1.61-2.98) |  |  | 1.67(1.22-2.29) |  |  | 1.59(1.09-2.30) |  |
| Marital status |  | <0.001 |  |  | 0.570 |  |  | 0.579 |
| Single | Reference |  |  | Reference |  |  | Reference |  |
| Married | 1.64(1.11-2.42) |  |  | 0.90(0.66-1.25) |  |  | 0.83(0.51-1.35) |  |
| Other | 2.17(1.40-3.38) |  |  | 0.92(0.65-1.32) |  |  | 0.75(0.43-1.29) |  |
| Poverty Rate§ |  | 0.003 |  |  | 0.003 |  |  | 0.034 |
| ≤Quartile 1 (6.49%) | Reference |  |  | Reference |  |  | Reference |  |
| ≤Quartile 2 (9.15%) | 1.14(0.77-1.70) |  |  | 1.24(0.83-1.85) |  |  | 1.08(0.64-1.83) |  |
| ≤Quartile 3 (13.15%) | 0.96(0.65-1.44) |  |  | 0.93(0.62-1.39) |  |  | 0.83(0.50-1.38) |  |
| >Quartile 3 (13.15%) | 1.76(1.21-2.55) |  |  | 1.74(1.19-2.55) |  |  | 1.54(0.95-2.52) |  |

† Full model multivariable cox regression analysis after propensity score matching.

§ All data are county level.

Table S4: Histologic Subtypes of Secondary Malignancies After Early-stage PB-DLBCL

| SPM site | Histologic Subtypes | Number of patients |
| --- | --- | --- |
| Oral cavity and pharynx | Squamous cell carcinoma | 1 |
| Digestive System | Adenocarcinoma | 8 |
|  | Hepatocellular carcinoma | 1 |
| Respiratory System | Squamous cell carcinoma | 2 |
|  | Acinar cell carcinoma | 1 |
|  | Adenocarcinoma | 2 |
|  | Non-small cell carcinoma | 1 |
|  | Neoplasm, malignant | 1 |
| Bones and Joints | Chondrosarcoma | 1 |
| Soft Tissue including Heart | Malignant fibrous histiocytoma | 1 |
|  | Giant cell sarcoma | 1 |
| Female Breast | Infiltrating duct carcinoma | 5 |
| Female Genital System | Adenocarcinoma | 1 |
|  | Mixed cell adenocarcinoma | 1 |
| Male Genital System | Adenocarcinoma | 8 |
|  | Basal cell carcinoma | 1 |
| Urinary System | Clear cell adenocarcinoma | 2 |
|  | Papillary transitional cell carcinoma | 2 |
|  | Transitional cell carcinoma | 1 |
| Brain and Other Nervous System | Gliosarcoma | 1 |
| Endocrine System | Papillary adenocarcinoma | 3 |
| Non-Hodgkin Lymphoma | Diffuse large B-cell lymphoma | 1 |
|  | Follicular lymphoma | 1 |
|  | Extranodal marginal zone lymphoma of mucosal-assoc. lymphoid tissue-MALT | 1 |
|  | Primary cutaneous T-cell lymphoma | 1 |
|  | Non-Hodgkin lymphoma, NOS | 1 |
| Acute Myeloid Leukemia | Therapy-related myeloid neoplasm | 2 |
| Melanoma of the Skin | Malignant melanoma | 1 |
|  | Lentigo maligna melanoma | 1 |

Abbreviations: SPM, second primary malignancies; NOS, not otherwise specified.

Table S5: Secondary Cancers with Elevated Risk After Early-stage PB-DLBCL Stratified by Age.

| Age, y | 18-39 | | | | 40-64 | | | | 65+ | | | |
| --- | --- | --- | --- | --- | --- | --- | --- | --- | --- | --- | --- | --- |
| Treatment | Chemotherapy alone | | Combined modality therapy | | Chemotherapy alone | | Combined modality therapy | | Chemotherapy alone | | Combined modality therapy | |
| SPM site† | O | SIR (95% CI) | O | SIR (95% CI) | O | SIR (95% CI) | O | SIR (95% CI) | O | SIR (95% CI) | O | SIR (95% CI) |
| All sites | 1 | 1.02(0.03-5.71) | 8 | 3.52*(1.52-6.94) | 8 | 1.22(0.53-2.4) | 24 | 1.15(0.74-1.72) | 5 | 0.74(0.24-1.73) | 26 | 1.28(0.83-1.87) |
| Cancer of the Tonsil | 0 | 0(0-569.89) | 0 | 0(0-189.79) | 0 | 0(0-129.44) | 2 | 18.57*(2.25-67.09) | 0 | 0(0-380.21) | 0 | 0(0-118.86) |
| Cancer of the Ascending Colon | 0 | 0(0-600.07) | 1 | 67.31*(1.7-375.03) | 0 | 0(0-50.98) | 0 | 0(0-17.08) | 0 | 0(0-28.15) | 2 | 5.19(0.63-18.76) |
| Cancer of the Respiratory System | 0 | 0(0-48.47) | 3 | 19.06*(3.93-55.7) | 0 | 0(0-3.6) | 3 | 0.97(0.2-2.83) | 0 | 0(0-3.19) | 6 | 1.77(0.65-3.85) |
| Cancer of the Bones and Joints | 0 | 0(0-564.11) | 1 | 120.99*(3.06-674.11) | 0 | 0(0-687.42) | 0 | 0(0-175.28) | 0 | 0(0-687.42) | 0 | 0(0-222.04) |
| Cancer of Cervix Uteri | 1 | 49.92*(1.26-278.11) | 0 | 0(0-107.13) | 0 | 0(0-161.95) | 0 | 0(0-60.92) | 0 | 0(0-215.41) | 0 | 0(0-68.21) |

Abbreviations: SPM, second primary malignancies; O: Observed Cases; SIR: Standardized Incidence Ratios; CI, Confidence interval.

* P < 0.05

† Only sites with significant SIR (P<0.05) are shown.

Table S6: Secondary Cancers with Elevated Risk After Early-stage PB-DLBCL Stratified by Primary Site of Bone Involvement.

| Primary Site | Appendicular bones | | | | Axial bones | | | |
| --- | --- | --- | --- | --- | --- | --- | --- | --- |
| Treatment | Chemotherapy alone | | Combined modality therapy | | Chemotherapy alone | | Combined modality therapy | |
| SPM site† | O | SIR (95% CI) | O | SIR (95% CI) | O | SIR (95% CI) | O | SIR (95% CI) |
| All sites | 4 | 0.85(0.23-2.18) | 28 | 1.59*(1.06-2.3) | 10 | 1.05(0.5-1.92) | 30 | 1.16(0.78-1.66) |
| Cancer of the Bones and Joints | 0 | 0(0-639.87) | 1 | 48.03*(1.22-267.6) | 0 | 0(0-393.36) | 0 | 0(0-146.94) |
| Cancer of the Uterus | 0 | 0(0-1169.23) | 1 | 90.30*(2.29-503.11) | 0 | 0(0-527.78) | 0 | 0(0-261.73) |
| All Lymphatic and Hematopoietic Diseases | 0 | 0(0-8.09) | 5 | 3.10*(1.01-7.23) | 2 | 2.31(0.28-8.34) | 2 | 0.84(0.1-3.05) |

Abbreviations: SPM, second primary malignancies; O: Observed Cases; SIR: Standardized Incidence Ratios; CI, Confidence interval.

* P < 0.05

† Only sites with significant SIR (P<0.05) are shown.

Table S7: Patient Characteristics and Bias for Radiotherapy in Advanced-stage Patients.

| Characteristic | Total | Chemotherapy alone | Combined modality therapy | P* |
| --- | --- | --- | --- | --- |
|  | 464(100%) | 268(57.8%) | 196(42.2%) |  |
| Age, y |  |  |  | 0.179 |
| 18-39 | 79(17.0%) | 52(19.4%) | 27(13.8%) |  |
| 40-64 | 165(35.6%) | 88(32.8%) | 77(39.3%) |  |
| 65+ | 220(47.4%) | 128(47.8%) | 92(46.9%) |  |
| Sex |  |  |  | 0.830 |
| Male | 256(55.2%) | 149(55.6%) | 107(54.6%) |  |
| Female | 208(44.8%) | 119(44.4%) | 89(45.4%) |  |
| Year of diagnosis |  |  |  | 0.304 |
| 2002-2009 | 238(51.3%) | 132(49.3%) | 106(54.1%) |  |
| 2010-2016 | 226(48.7%) | 136(50.7%) | 90(45.9%) |  |
| Race |  |  |  | 0.378 |
| White | 408(87.9%) | 236(88.1%) | 172(87.8%) |  |
| Black | 29(6.3%) | 14(5.2%) | 15(7.7%) |  |
| Other | 27(5.8%) | 18(6.7%) | 9(4.6%) |  |
| Stage |  |  |  | 0.522 |
| III | 27(5.8%) | 14(5.2%) | 13(6.6%) |  |
| IV | 437(94.2%) | 254(94.8%) | 183(93.4%) |  |
| Primary Site |  |  |  | 0.500 |
| Appendicular | 139(30.0%) | 77(28.7%) | 62(31.6%) |  |
| Axial | 325(70.0%) | 191(71.3%) | 134(68.4%) |  |
| Marital status |  |  |  | 0.253 |
| Single | 77(16.6%) | 48(17.9%) | 29(14.8%) |  |
| Married | 261(56.3%) | 142(53.0%) | 119(60.7%) |  |
| Other | 126(27.2%) | 78(29.1%) | 48(24.5%) |  |
| Poverty Rate§ |  |  |  | 0.675 |
| ≤Quartile 1 (6.49%) | 116(25.0%) | 70(26.1%) | 46(23.5%) |  |
| ≤Quartile 2 (9.15%) | 120(25.9%) | 72(26.9%) | 48(24.5%) |  |
| ≤Quartile 3 (13.15%) | 138(29.7%) | 74(27.6%) | 64(32.7%) |  |
| >Quartile 3 (13.15%) | 90(19.4%) | 52(19.4%) | 38(19.4%) |  |

* P-value from chi-square tests.
§ All data are county level.

Table S8: Prognostic Factors for Overall Survival in Advanced-stage Patients.

|  | Univariate | |  | Multivariate | |
| --- | --- | --- | --- | --- | --- |
|  | HR (95% CI) | P |  | HR (95% CI) | P |
| Treatment |  | 0.777 |  |  | 0.922 |
| Chemotherapy alone | Reference |  |  | Reference |  |
| Combined modality therapy | 1.04(0.78-1.40) |  |  | 0.99(0.73-1.33) |  |
| Age, y |  | <0.001 |  |  | <0.001 |
| 18-39 | Reference |  |  | Reference |  |
| 40-64 | 4.75(2.04-11.07) |  |  | 4.41(1.86-10.43) |  |
| 65+ | 10.37(4.56-23.57) |  |  | 9.59(4.12-22.34) |  |
| Sex |  | 0.956 |  |  |  |
| Male | Reference |  |  | - |  |
| Female | 0.99(0.74-1.33) |  |  | - |  |
| Year of diagnosis |  | 0.353 |  |  |  |
| 2002-2009 | Reference |  |  | - |  |
| 2010-2016 | 0.86(0.62-1.19) |  |  | - |  |
| Race |  | 0.966 |  |  |  |
| White | Reference |  |  | - |  |
| Black | 0.99(0.55-1.78) |  |  | - |  |
| Other | 1.08(0.59-2.00) |  |  | - |  |
| Stage |  | 0.836 |  |  |  |
| III | Reference |  |  | - |  |
| IV | 1.07(0.56-2.03) |  |  | - |  |
| Primary Site |  | 0.011 |  |  | 0.042 |
| Appendicular | Reference |  |  | Reference |  |
| Axial | 1.57(1.11-2.23) |  |  | 1.44(1.01-2.04) |  |
| Marital status |  | 0.012 |  |  | 0.751 |
| Single | Reference |  |  | Reference |  |
| Married | 1.84(1.12-3.03) |  |  | 1.03(0.61-1.72) |  |
| Other | 2.26(1.32-3.87) |  |  | 1.16(0.67-2.02) |  |
| Poverty Rate§ |  | 0.350 |  |  |  |
| ≤Quartile 1 (6.49%) | Reference |  |  | - |  |
| ≤Quartile 2 (9.15%) | 0.96(0.63-1.48) |  |  | - |  |
| ≤Quartile 3 (13.15%) | 1.29(0.87-1.92) |  |  | - |  |
| >Quartile 3 (13.15%) | 1.28(0.82-1.98) |  |  | - |  |

§ All data are county level.

**Figure S1.** The calibration curves for predictions of overall survival in the training cohort (A-C) and the validation cohort (D-F) at 3, 5, and 10 years after diagnosis;

**Figure S2**. Kaplan–Meier curves depicting overall survival by treatment group after propensity score matching in stage I-II. CMT, combined modality therapy.
