# Supplementary figures and images for: Role of Radiation Therapy Differs Between Stages in Primary Bone Large B-Cell Lymphoma in Rituximab Era: A Population-Based Analysis
Source: Front Oncol. 2020 Jul 14;10:1157. doi: 10.3389/fonc.2020.01157 (PMC7372636; doi:10.3389/fonc.2020.01157)

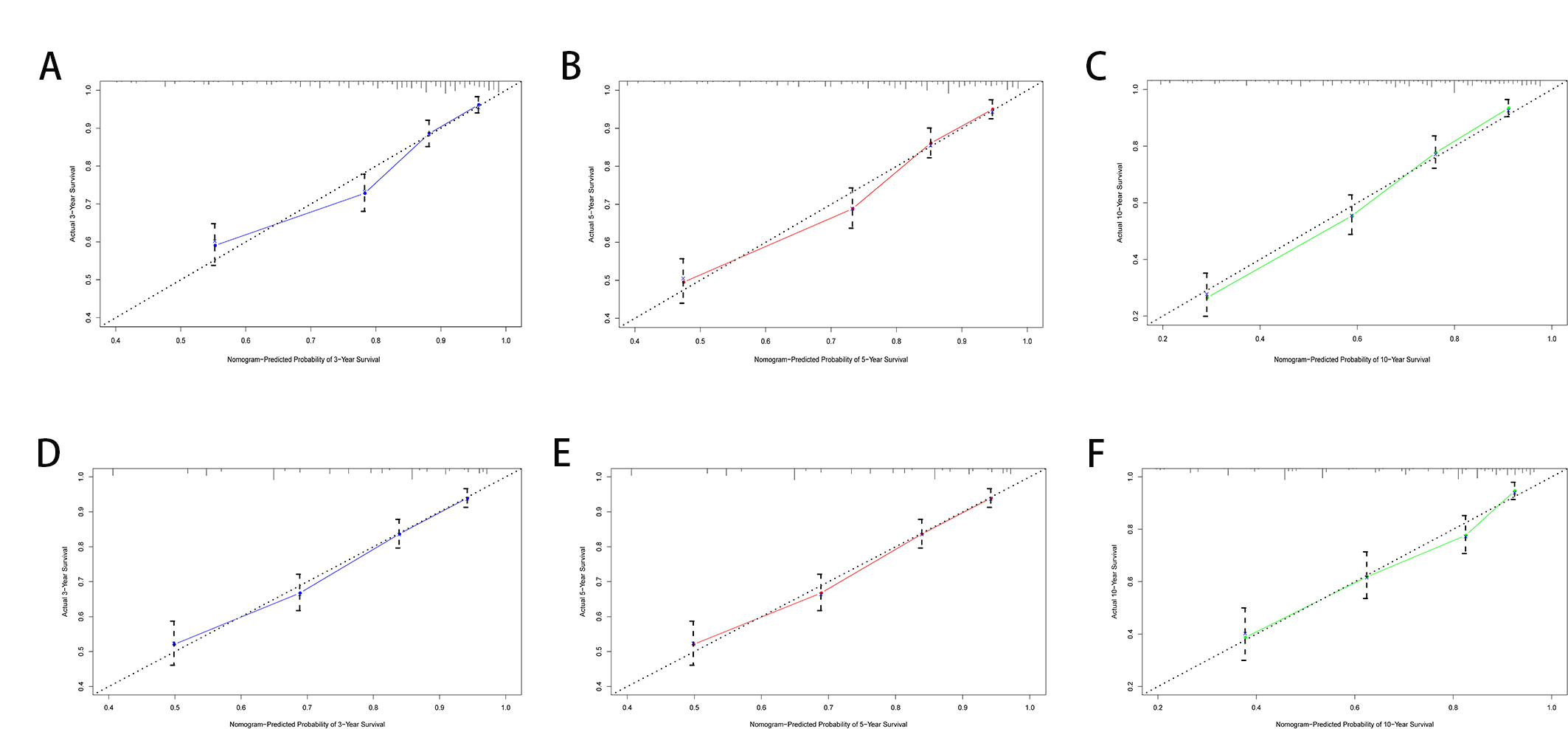

Supplement: Supplementary file 2 [file Image_1.jpeg]

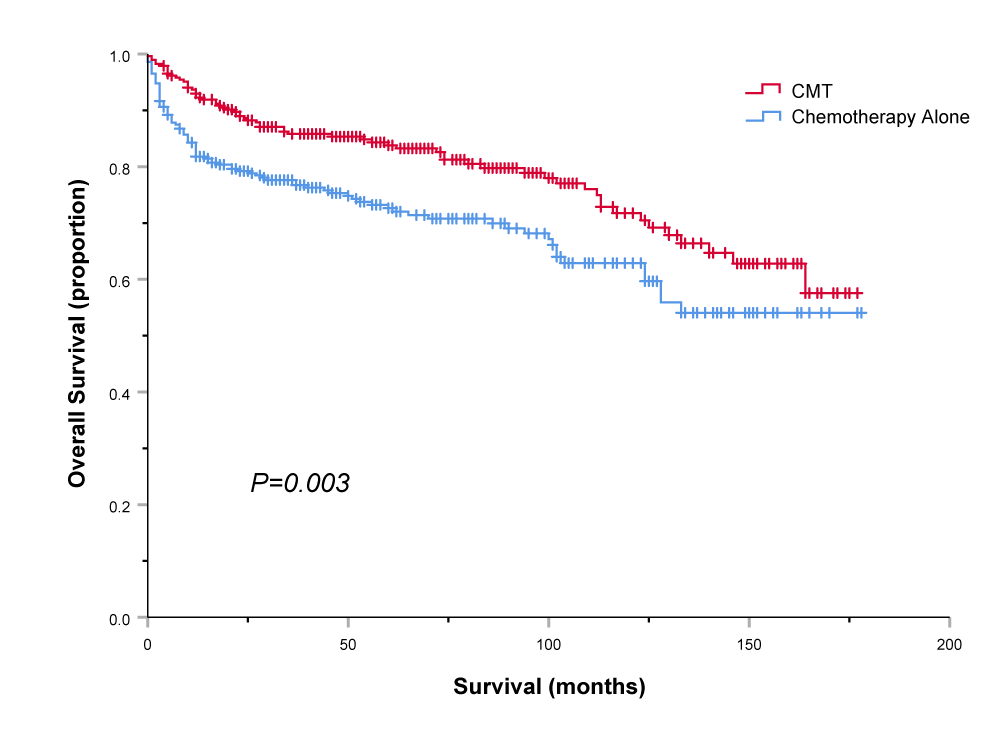

Supplement: Supplementary file 3 [file Image_2.tif]
